# Utilization of Targeted RNA-Seq for the Resolution of Variant Pathogenicity and Enhancement of Diagnostic Yield in Dysferlinopathy

**DOI:** 10.3390/jpm13030520

**Published:** 2023-03-13

**Authors:** Laura Rufibach, Kiera Berger, Samya Chakravorty, Sarah Emmons, Laurie Long, Greg Gibson, Madhuri Hegde

**Affiliations:** 1 Jain Foundation, Inc., Seattle, WA 98115, USA; sshira@jain-foundation.org (S.E.); llong@jain-foundation.org (L.L.); 2 Center for Integrative Genomics, Georgia Institute of Technology, Atlanta, GA 30332, USA; kieraberger07@gmail.com (K.B.); ggibson.gt@gmail.com (G.G.); 3 Department of Human Genetics, Emory University School of Medicine, Atlanta, GA 30322, USA; samya.rajrup@gmail.com (S.C.); madhuri.hegde@perkinelmer.com (M.H.); 4 Department of Pediatrics, Emory University School of Medicine, Atlanta, GA 30322, USA; 5 PerkinElmer Genomics, Global Laboratory Services, Waltham, MA 02451, USA

**Keywords:** RNA-Seq, dysferlinopathy, LGMD, variants of unknown significance, genetic diagnosis, ACMG guidelines, LGMDR2, LGMD2B, Miyoshi myopathy, diagnostic yield

## Abstract

For inherited diseases, obtaining a definitive diagnosis is critical for proper disease management, family planning, and participation in clinical trials. This can be challenging for dysferlinopathy due to the significant clinical overlap between the 30+ subtypes of limb–girdle muscular dystrophy (LGMD) and the large number of variants of unknown significance (VUSs) that are identified in the dysferlin gene, *DYSF*. We performed targeted RNA-Seq using a custom gene-panel in 77 individuals with a clinical/genetic suspicion of dysferlinopathy and evaluated all 111 identified *DYSF* variants according to the American College of Medical Genetics and Genomics and the Association for Molecular Pathology (ACMG/AMP) guidelines. This evaluation identified 11 novel *DYSF* variants and allowed for the classification of 87 *DYSF* variants as pathogenic/likely pathogenic, 8 likely benign, while 16 variants remained VUSs. By the end of the study, 60 of the 77 cases had a definitive diagnosis of dysferlinopathy, which was a 47% increase in diagnostic yield over the rate at study onset. This data shows the ability of RNA-Seq to assist in variant pathogenicity classification and diagnosis of dysferlinopathy and is, therefore, a type of analysis that should be considered when DNA-based genetic analysis is not sufficient to provide a definitive diagnosis.

## 1. Introduction

Dysferlinopathy is an autosomal recessively inherited muscular dystrophy caused by variants in the *DYSF* gene (OMIM: 603009) that predominantly affects skeletal muscle and results in progressive muscle weakness and wasting [1,2,3]. Dysferlin is a large transmembrane protein (237 kDA) that is highly expressed in the skeletal muscles [4]. Studies have implicated dysferlin in multiple roles in skeletal muscle including membrane repair [5], T-tubule structure and function [6], vesicle trafficking [7], endocytosis [8], and lipid metabolism [9,10]. However, the function or functions that play the most prominent role in the disease pathogenesis have yet to be elucidated.

The most common clinical diagnoses associated with dysferlinopathy are limb–girdle muscular dystrophy R2 dysferlin related (formerly LGMD2B) and Miyoshi myopathy (MMD1) [11,12,13]. LGMDR2 describes a phenotype that consists predominantly of proximal limb weakness at presentation [14], while MMD1 is associated with predominantly distal limb weakness at presentation [15]. However, the natural history analysis of dysferlinopathy has shown that there are no clinically relevant differences between the presenting phenotypes, which clarifies that they are the same disease (i.e., dysferlinopathy) [16]. There are currently no treatments for dysferlinopathy.

Dysferlinopathy is part of a larger group of muscular dystrophies classified as limb–girdle muscular dystrophies (LGMDs). The LGMDs are one of the most prevalent and heterogeneous inherited group of neuromuscular disorders (NMDs), with more than 30 monogenic clinically overlapping subtypes [12]. This clinical overlap makes it difficult to reach a definitive diagnosis exclusively based on clinical phenotype, therefore genetic analysis is required in order to differentiate between the various LGMD subtypes, as well as other muscular dystrophies. Despite the greater accuracy, availability, and lower cost for DNA genetic sequencing that has been achieved, obtaining a definitive genetic diagnosis still remains challenging. Recently, in a large LGMD 35 gene panel next generation DNA sequencing (NGS) program, only 27% of participants (1259 out of 4656 patients) received a definitive diagnosis based on the identification of pathogenic variants [17]. In addition, 72% of all clinically reportable variants in this study were variants of unknown significance (VUS), resulting in ~50% of all patients, including at least 90 patients with *DYSF* VUSs or unresolved compound heterozygosity, remaining undiagnosed [17]. With upcoming trials of dysferlin dual AAV gene therapy [18] and other possible therapeutics such as calcium regulators [19], chemical chaperones [20] and cholesterol/lipid modulators [9,21] being evaluated as possible treatments for dysferlinopathy and the fact that definitive molecular diagnosis is typically a prerequisite for participation in clinical trials, it is critical to improve our understanding and classification of *DYSF* VUSs so that more individuals can obtain a definitive diagnosis and be able to participate in clinical trials.

Rigorous reclassification of VUSs per American College of Medical Genetics and Genomics (ACMG) guidelines [22] requires understanding disease mechanisms using an integrative approach that combines functional assays with phenotype correlation [23,24,25,26]. For NMDs, the use of affected tissue (muscle) or skin derived transdifferentiated myotubes is ideal; however, biopsies are invasive, painful, costly, difficult to obtain, and quality can be compromised by adipocyte contamination. Hence, minimally invasive gene based testing or evaluation of other biomarkers from an easily accessible tissue (e.g., blood/urine) is needed. It is known that DYSF is highly expressed in blood monocytes [27] and we and others have previously shown that DYSF protein expression in blood is a highly effective biomarker for dysferlinopathy [28,29,30,31]. However, protein evaluation by itself is not able to provide a definitive diagnosis, nor is it conclusive with regards to reclassifying VUSs. Therefore, other genetic based analyses are needed. The use of NGS-based transcriptome sequencing (RNA-Seq) using patient muscle biopsies, myotubes, or fibroblasts has been used by us and others to increase the diagnostic yield in NMDs [32,33] and mitochondriopathies [34]. Notably, Frésard et al. [35] achieved a 24.2% diagnostic yield in a clinically diverse cohort after performing whole transcriptome sequencing from blood.

**Figure 1:**
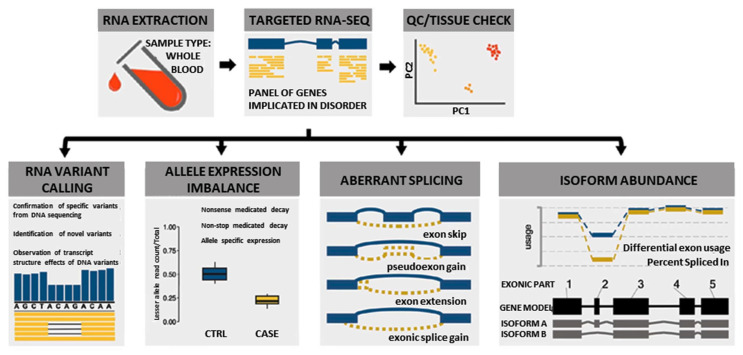
Overview of multi-faceted approach used to analyze RNA to aid in the diagnosis of dysferlinopathy.

In this study, we used a custom blood-based targeted 274 NMD gene panel RNA-Seq analysis (Supplementary Table S1) to identify novel pathogenic variants and elucidate pathogenic disease mechanisms by evaluating splicing, allelic expression, and exon usage (Figure 1). This information was combined with clinical, protein, and other relevant data using the ACMG/AMP guidelines to classify or reclassify the pathogenicity of the 111 *DYSF* variants identified in the cohort of 77 individuals evaluated. This robust classification of *DYSF* variants led to a significant increase in the diagnostic yield of this subset of individuals. Our results illustrate the importance of performing further functional genomic analysis when DNA based analysis is not sufficient to provide a definitive diagnosis.

## 2. Materials and Methods

### 2.1. Patient Recruitment

A total of 77 individuals with a clinical and/or genetic suspicion of dysferlinopathy or a closely related LGMD but without a definitive molecular diagnosis were recruited for the RNA-Seq study at Emory University between 2017–2020. These specific 77 individuals were chosen because it was determined that RNA-Seq may be able to help with the clarification of their diagnosis through the identification of *DYSF* variants and/or by providing information such as allelic phasing or proof of aberrant splicing that could potentially lead to a pathogenic variant classification. Fifty-six (56) individuals for this study were recruited with the help of the Jain Foundation (cases labeled with JF prior to their ID number in Supplementary Table S2). These individuals contacted the foundation for diagnostic support and then were referred to this study. Written informed consents were obtained from all the JF participants according to the Emory Institutional Review Board approval (IRB00055448). Thirty (30) cases were recruited through the Clinical Outcome Study for dysferlinopathy (COS), including 9 samples that overlapped with 9 of the JF samples. For the COS cases (cases labeled with a C prior to their ID number in Supplementary Table S2), the de-identified RNA and DNA used in the study was obtained from the Eurobiobank in Newcastle, UK. These samples were given to the biobank after proper consent was obtained as part of COS [36] and transferred to this study using a material transfer agreement between institutions. In addition to the 77 patient samples, 15 control individuals, who were verified to express normal DYSF protein levels, were also recruited after giving informed consent.

### 2.2. Clinical Evaluation

All patients underwent comprehensive clinical evaluation by their respective physicians and dysferlinopathy or a closely related LGMD was suspected. Clinical history including age of onset, initial symptoms, region in which weakness first started, functional status, and pattern of weakness was collected where detailed clinical notes were available. For any participant who agreed, the patient and/or their physician were asked a full list of relevant questions (see Supplementary Materials and Methods “Patient Enrollment Questionnaire” section) regarding their clinical symptoms and clinical and family history in order to assess the patient’s phenotype through the running of the automated LGMD diagnostic assistant (ALDA) algorithm. ALDA was developed by the Jain Foundation to predict up to the 3 most probable LGMD subtypes based on the clinical features of each patient (https://jain-foundation-alda.org/node/1) [17,31]. DYSF protein expression information from muscle biopsies and/or evaluation of blood monocytes were obtained where available. Genotype information from prior CLIA-CAP certified genetic testing reports was also collected. These genetic tests were heterogeneous, ranging from exome or array comparative genomics hybridization to known mutation Sanger sequencing, based on their physician’s discretion. All clinical information obtained is listed in Supplementary Table S3.

### 2.3. Targeted RNA-Seq Library Preparation and Sequencing from Whole Blood

High quality (RNA integrity number; RIN > 7) RNA was extracted from whole blood of the patients and control individuals using QIAamp RNA blood kit (cat # 52304, Qiagen, Germantown, MD, USA) following the manufacturer’s protocol. Only blood specimens in EDTA tubes shipped to us within 24 h from a blood draw based on the time log on top of the EDTA vial were used for RNA extraction to control for any RNA degradation effect. Library preparation was performed using Agilent SureSelectXT RNA target enrichment kit for Illumina multiplexed sequencing (cat# G9691-9000, Agilent Technologies, Santa Clara, CA, USA) following manufacturer’s protocol. Targeted RNA-Seq was performed in 73 of the cases to have a more focused clinically relevant platform for NMD diagnostics and to achieve greater read depth and coverage of the target NMD genes. We used a custom-designed target library probe to capture 274 genes (listed in Supplementary Table S1) that are known to be NMD-associated and are known to have skeletal muscle expression as retrieved from The Genotype-Tissue Expression (GTEx) portal [37]. For the remaining 4 cases (JF194, JF173, JF392, JF54), whole mRNA sequencing was performed. Strand-specific paired-end 150 bp sequencing was performed on an Illumina NextSeq instrument to obtain output at a depth of more than 15 million reads per sample.

### 2.4. Bioinformatics Workflow

We used a novel multi-faceted approach of targeted RNA-Seq analysis for all cases, which included the evaluation of RNA variant calling, allele expression imbalance, aberrant splicing, and isoform abundance (Figure 1). Together the results of the RNA-Seq analysis, available clinical information, and DYSF protein levels were used to aid in variant classification and diagnostic clarification. All RNA-Seq assay results were reported back to patients and/or their respective physicians as research reports (not a diagnostic report) according to the guidelines of the approved Institutional Review Board protocol. Detailed methods of RNA-Seq data alignment and quality control, variant calling, splicing, allele expression imbalance, and gene expression analysis in mRNA are described in the Supplementary Materials and Methods. Code for performing targeted RNA-Seq is available on our GitHub account at https://github.com/kiera-gt/rnaseq-nmd.

### 2.5. Additional DNA Based Sequencing

Whenever a new variant was identified by RNA-Seq that was not identified by the DNA analysis previously performed, the necessary DNA analysis (whole genome sequencing (WGS), targeted NGS DNA testing of a 131 gene NMD gene panel, copy number variation (CNV) analysis for the identification of exonic deletions or duplications, and/or Sanger sequencing) was performed by Perkin Elmer, a CLIA-CAP certified facility, to confirm the presence and/or identify of the DNA variant.

### 2.6. Variant Reclassification

*DYSF* reference transcript NM_003494.4 was used for all analyses, noting that, in blood transcripts, *DYSF* exon 17 is mostly spliced out and that transcripts containing the alternative exons 5a (NM_001130980) and 40a (NM_001130981) are sometimes present. The pathogenicity of the 111 *DYSF* variants identified in the individuals in this study were evaluated according to the standard ACMG/AMP criteria [22], with a few modifications. The full list of the relevant ACMG/AMP codes used for the pathogenicity classification are described in the Supplementary Materials and Methods section. The variants analyzed in this study and the evidence used for variant classification are provided in Supplementary Table S4.

## 3. Results

RNA-Seq was performed on RNA isolated from the blood samples of 77 individuals suspected to have dysferlinopathy or who had an incomplete genetic diagnosis (Supplementary Table S2). RNA-Seq has the ability to provide many different data points that can aid in the identification and clarification of variants, which can help to finalize a diagnosis (Figure 1). These include identification of splicing errors, potential structural variants (e.g., intragenic insertion–deletion events), and new variants, as well as determination of allelic phasing and inference of nonsense mediated decay (nmd) from allelic imbalance.

### 3.1. Determination of Allelic Phasing of Variants to Aid in Determination of Pathogenicity

**Figure 2:**
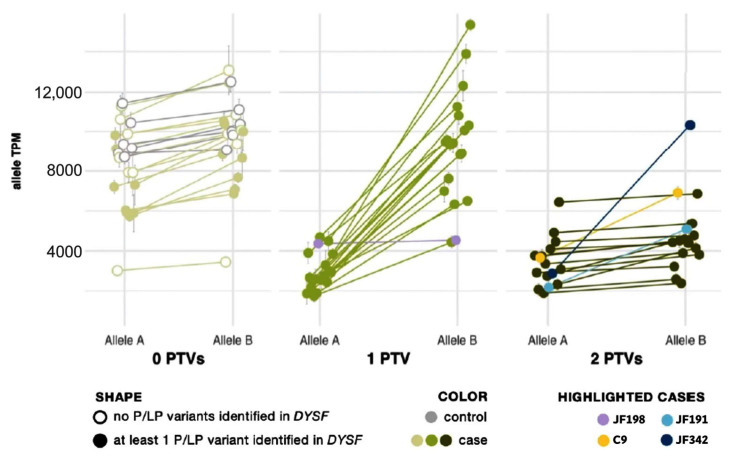
Allele expression imbalance caused by nonsense-mediated decay of transcripts containing a protein truncating variant (PTV). Samples are grouped by the number of PTVs observed in *DYSF* mRNA. See Supplemental Material and Methods for calculation details. In each sample, the average percent and standard deviation were taken for Allele A and Allele B and mapped onto the sample’s overall gene abundance to estimate the abundance of each *DYSF* allele copy. Cases with one PTV (medium green dots) show normal expression of Allele B and reduced expression of Allele A (a result of nonsense-mediated decay acting upon the transcript copy containing the PTV). Cases with 2 PTVs (dark green dots) show a reduction in both copies and a ratio of Allele A to Allele B similar to cases with 0 PTVs (light green dots). Highlighted cases (lavender, yellow, teal, navy) are examples of the need to use caution in the interpretation of AEI (see text for details).

Protein-truncating variants (PTVs) such as nonsense and frameshift variants are commonly subject to nmd to prevent accumulation or protein translation of a nonfunctional or potentially deleterious transcript [38]. In previous studies, nmd has prevented some PTVs from being called in mRNA [39]. However, the extremely high read depth afforded by our targeted RNA-Seq panel, even without using any nmd inhibitor, allowed us to not only confidently call PTVs but also to use their presence to phase *DYSF* variants and confirm that two potentially causative variants are in trans without sequencing parents or offspring. As shown by Cummings et al. [32], the greater:lesser allele ratio in genes where a sample had one truncating single nucleotide variant (SNV) tends to be ~75:25. We observed the same trend in SNVs. We correlated this ratio with an overall transcript abundance of *DYSF*, showing that the differences in transcript abundance among these samples is predominantly a result of nonsense-mediated decay (Figure 2). The allele expression imbalance in samples with one truncating variant was so consistent across exons and samples that it could often be reliably used to phase variants.

Allelic expression imbalance (AEI) analysis was able to phase *DYSF* variants in 20 cases that had one PTV (see yellow highlighting in the “Phasing determined by AEI ratios” column in Supplementary Table S2). Within each sample, the allele ratio was found to be consistent for SNVs across the *DYSF* gene. When SNVs were grouped by number of PTVs found in *DYSF*, we found that the lesser-expressed nucleotide expression (lesser allele expression) in patients with one PTV was significantly reduced to ∼25% (*p* = 7 × 10^−13^) as a result of nmd. In patients with biallelic PTVs we cannot phase the individual SNV because both transcripts are subject to nmd and, therefore, the SNV allele ratio generally returned to ~0.5, as seen in cases with 0 PTVs (Figure 2).

There are some caveats to using RNA-Seq data for variant phasing. Deletions/insertions, splice events, and variants in variably expressed exons somewhat follow the same pattern but with less accuracy due to known issues in reliable mapping/calling these variants. This can be seen in cases JF342 and JF191 (highlighted in navy and teal, respectively in Figure 2), in which one of the identified variants was found in the alternatively spliced *DYSF* exon 17. In addition, PTVs that were found late in the gene may not lead to nmd, which may be the cause of the nonconforming pattern for case JF198 (highlighted in lavender in Figure 2), which had a PTV in the last exon of *DYSF*. Finally, when a case inexplicably deviates from the observed pattern (C9, highlighted in yellow in Figure 2), the determination that the variants are in trans cannot reliably be made.

Phasing of variants was also possible using different methods in an additional four cases. In cases C14 and C7, the two *DYSF* variants affected the same exon (exon 26 in C14 and exon 23 in C7), thus the fact that no reference sequence was detected indicates that the two variants were in trans. For C188, the c.3444T>A and c.3445G>A were detected in the same RNA transcript, which indicated that these 2 variants were in cis. Lastly, parental testing was performed in JF358 and showed that the c.5181delA and c.1668_1669insGTT variants were in trans. This phasing information aided in the ACMG/AMP classification for 26 *DYSF* variants (see explanations in PM3 column of Supplementary Table S4)

### 3.2. Identification of Aberrant Splicing Variants

**Figure 3:**
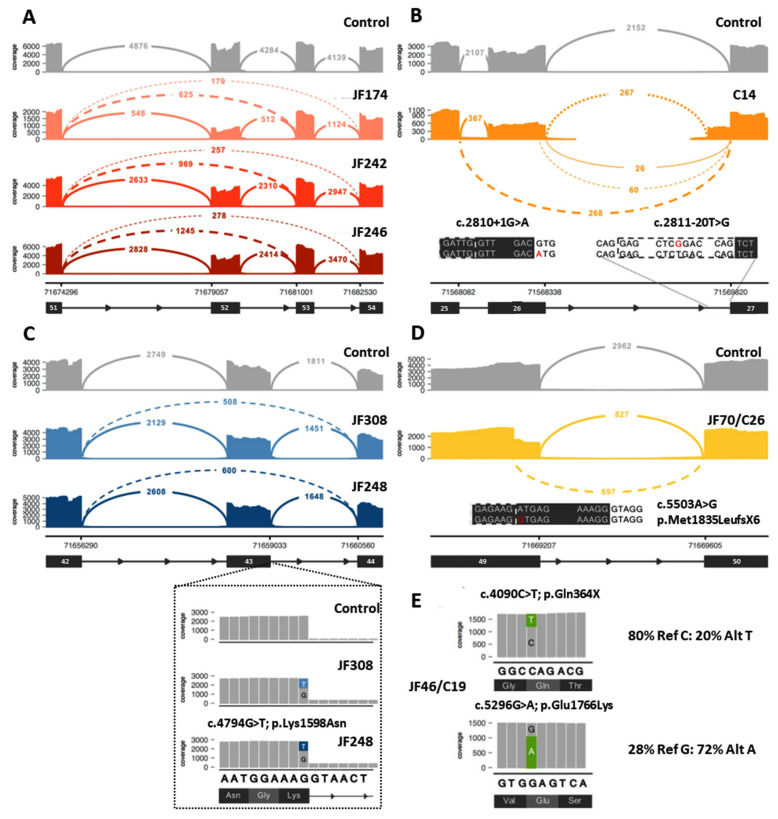
Examples of data that can be obtained from RNA-Seq analysis to aid in the diagnosis of dysferlinopathy. (**A**) Example of identification of exonic deletion: Sashimi plot of the exon skipping events seen in three patients (JF174, JF242, JF246). Subsequent genome sequencing (GS) identified the cause of the 2 different splicing events as a gross deletion encompassing exon 52. (**B**) Example of aberrant splicing events caused by intronic variants: RNA-Seq of C14 identified 2 different aberrant splicing events involving exon 26 caused by the destruction of the splice donor site by the c.2810+1G>A variant (left inset, red A indicates mutant variant) on one allele and the exon 27 extension caused by a novel branch point variant c.2811-20T>G (right inset, red G indicates mutant variant) on the other allele. (**C**) Example of leaky aberrant splicing event. Upper: Sashimi plot showing exon skipping caused by a leaky splice variant (c.4794G>T) at the end of exon 43 in JF308 and JF248. Inset: variant expression in a minority of reads (15–18%), which show a normal splice pattern but the presence of the missense variant (c.4794G>T; p.Lys1598Asn). (**D**) Example of missense variant acting as a splice altering variant: Sashimi plot of cryptic splice site variant c.5503A>G (inset, red G indicates mutant variant) in JF70/C26 that leads to a premature stop codon (p.Met1835LeufsX6) in the transcript. (**E**) Example of allele expression imbalance (AEI) observed in read pile-ups for JF46/C19 that can be used to confirm that the two variants of interest are in trans. In Sashimi plots seen in (**A**) through (**D**), solid lines indicate reference splicing, while dashed lines show observed aberrant splicing and values indicate the number of spliced transcripts.

Twenty-seven (27) *DYSF* variants were shown to cause aberrant splicing (Table 1, Supplementary Table S2). Twenty-two (22) of these variants were located within an intron, while 5 of the variants (c.857T>A, c.3031G>C, c.4794G>T, c.5429G>A, c.5503A>G, highlighted in gray in Table 1, Figure 3C,D) were exonic and originally predicted to be missense variants. Two of the exonic variants (c.4794G>T and c.857T>A) were leaky splice site variants that resulted in transcripts containing either the aberrant splicing event or the originally predicted missense variant. For c.4794G>T, 26–30% of the transcripts had the in frame deletion of exon 43, while another 15–18% of the transcripts contained the c.4794G>T; p.K1598N missense variant (Figure 3C). For c.857T>A, only 3–5% of the transcripts resulted in the in frame deletion of exon 9, while 50–60% of the transcripts contained the c.857T>A; p.Val286Glu missense variant.

Ten (10) of these splicing variants result in more than one splicing event. The majority of the splicing events result in small intragenic deletions that modify the reading frame and lead to premature translational termination, while a minority (6) lead to deletions that maintain the reading frame and allow for protein production. The transcript percentages identified for each aberrant splicing event are listed in Table 1. For many of the splicing events that lead to a frameshift, the transcript percentages do not add up to the expected 50% frequency for variants found in a compound heterozygous state. This is likely due to nmd of the RNA that contains the frameshifting variant that skews the percentages in favor of the allele that does not contain a frameshifting variant and is not undergoing nmd. However, when a splicing mutation that leads to a frameshift is paired with another frameshift or nonsense mutation on the other allele and both undergo nmd, than the transcript percentages are again closer to 50:50 because the RNA from both alleles is undergoing nmd. Three of the variants that led to aberrant splicing (c.1171_1180+4dup14, c.5429G>A, and c.5526-7T>G) were found in the homozygous state, resulting in transcript percentages that were at or close to the expected 100%. Transcript percentages were not possible to obtain for three of the splicing errors (c.1481-1G>A, c.2810+1G>A, and c.2811-20T>G). The reason percentages were not obtained for c.2810+1G>A and c.2811-20T>G was because these two *DYSF* variants are found in the same individual (C14) and, therefore, no normal reference sequence is present for this region, making it impossible to determine the exact percentages of the individual aberrant splicing events (Figure 3B). The c.1481-1G>A variant found in JF342 led to a 2 bp deletion in exon 17 and a frameshift due to the use of an alternate splice acceptor site in exon 17. In blood cells (compared with muscle) this aberrant splicing does not occur very often because the majority of the blood *DYSF* transcripts naturally splice out exon 17 [40] and therefore this variant is not observed in blood. Given the low frequency of the frameshifting event in blood, it was not possible to accurately assess the frequency of this splicing error.

### 3.3. Identification of New Variants by RNA-Seq

A total of 27 of the 77 RNA-Seq cases had either zero *DYSF* variants (JF371, JF392, JF340) or only one *DYSF* variant identified prior to the start of the study (JF362, JF203, JF277, JF368, JF174, JF246, JF242, JF369, C11, C14, C163, C7, JF313, C32, C8, C9, JF67, JF15, JF126, JF194, JF130, JF118, JF370, JF251). RNA-Seq led to the identification of additional pathogenic or likely pathogenic (P/LP) *DYSF* variants in 17 of these cases (JF362, JF203, JF277, JF368, JF174, JF246, JF242, JF369, C11, C14, C163, C7, JF313, C32, C8, C9, JF67), the identification of possible causative variants in other genes associated with other types of neuromuscular disease in 3 cases (JF15, JF126, JF371), and no additional *DYSF* variants in 7 cases (JF194, JF130, JF118, JF370, JF392, JF251, JF340) (Supplementary Table S2). In addition, a new *DYSF* variant was identified in an additional five cases that had two or more *DYSF* variants identified prior to study onset with one or more of the previously identified *DYSF* variants being labeled as a VUS or likely benign (LB) (C98, C24, C195, C144, JF56). A new P/LP *DYSF* variant was identified in four out of these five cases (C98, C24, C195 and C144) and two variants in *COL6A2* were found in JF56.

Of the 21 cases where an additional P/LP *DYSF* variant was identified, 8 of the cases had an intragenic CNV (JF362, JF203, JF277, JF368, JF174, JF246, JF242, JF369). RNA-Seq of these cases showed splicing errors (Figure 3A), and isoform abundance analysis (Figure 1) revealed differential exon usage, which pointed to the presence of an intragenic deletion or duplication of one or more *DYSF* exons as the cause of the splicing error. Whole genome sequencing (WGS) or CNV analysis was done to confirm the presence of the predicted *DYSF* deletion or duplication identified by RNA-Seq. Seven (7) of these were exonic deletions (deletion of *DYSF* exons 2–3, exon 4, exons 25–29, exon 34, or exon 52) and one was a large exonic duplication (duplication of *DYSF* exons 10–35). The deletions of exon 4 and exon 34 and the duplication of exons 10–35 were novel and the deletions of exons 2–3, 25–29, and exon 52 have been previously reported [41]. Each of these events were found in a single case, except for the deletion of exon 52, which was found in three separate cases (JF174, JF246, JF242—Figure 3A), and the deletion of exons 25–29, which was found by RNA-Seq in JF277 and previously reported in C176 [36]. Interestingly, the intronic breakpoints for the three exon 52 deletions were different in each case, indicating that the events occurred independently (Supplementary Table S2). For the remaining 13 cases in which an additional P/LP *DYSF* variant was identified, 7 were variants found within a single exon, 5 were intronic variants located within 20 bp of the end of the exon, and 1 was the skipping of *DYSF* exons 23 and 24 (the causative DNA variant was unable to be identified because DNA for this individual was not available).

### 3.4. Reclassification of DYSF Variants

A total of 111 *DYSF* variants were identified in the 77 cases evaluated during this study. Of these, 11 of the variants (c.125dup, c.2643+5G>A, c.2811-20T>G, c.3113G>C, c.3904_4410del, c.4509+11586dupG, c.4526T>G, c.5341G>A, c.3703_3843del, c.237_342del, c.907-2774_3873+827del; bolded variants in Table 2) were novel as determined by the fact that they were not listed in the three main *DYSF* variant databases (UMD-DYSF, DYSF LOVD, NIH ClinVar) or found through internet searches. All variants observed by DNA sequencing prior to study onset were confirmed in the mRNA by RNA-Seq. All 111 *DYSF* variants found in this study were classified using the ACMG/AMP criteria indicated in the Supplementary Materials and Methods, and the detailed evidence and codes used to classify each variant using these criteria are shown in Supplementary Table S4. Using this analysis, 59 of the variants were classified as pathogenic (P), 28 were classified as likely pathogenic (LP), 8 were classified as likely benign (LB), and 16 remained VUS (Table 2). Of the 59 variants that were called pathogenic in this study, other sources had previously called them pathogenic in 40 cases, likely pathogenic in 2 cases, or both P/LP in 12 cases (total of 54). The five new pathogenic variants identified were either novel, previously classified as a VUS, had conflicting calls in other sources, or had not been rated. Of the 28 variants identified as likely pathogenic, 7 were novel, other sources previously called them pathogenic (5) or VUSs (11), or they had conflicting calls (5). For the eight likely benign variants classified by this study, other sources had previously called three benign/likely benign, four had conflicting calls, and one was a VUS. Overall, for the 33 variants that were classified as VUSs or had conflicting calls prior to the study, 64% (21) were able to be reclassified as pathogenic (2), likely pathogenic (14), or likely benign (5). The majority (11 out of 16) of the variants that were not able to be reclassified in this study and remained VUSs were missense variants, which are harder to reclassify and for which RNA-Seq does not often provide additional information to help support pathogenicity calls. This information is shown in Table 2.

The biggest impact this study had on pathogenicity classification was with the 28 variants that were identified as likely pathogenic by this study. Of the likely pathogenic classified variants, 75% (21) had at least one entry from other sources that listed it as a VUS or were novel previously uncharacterized variants. The most significant factors that allowed for the establishment of a likely pathogenic classification were the evidence provided by RNA-Seq for aberrant splicing and the allelic phasing of variants using AEI, the in vitro assay that showed the detrimental effects caused by missense variants [20], and the identification of previously unreported cases that allowed for the assigning or upgrading of the strength for PM3 and/or PP4. The pathogenicity call was downgraded from what it was called by other sources for six variants (c.857T>A, c.1168G>A, c.3031G>C, 4886+1249G>T, 5429+1G>T, c.6216delC), due to the use of more stringent criteria for assigning PP4, PP1, and PP3, the lack of a sufficient number of other cases, and the lack of specific information about how the pathogenicity calls for variants listed in ClinVar were made.

### 3.5. Increase in Diagnostic Yield

**Figure 4:**
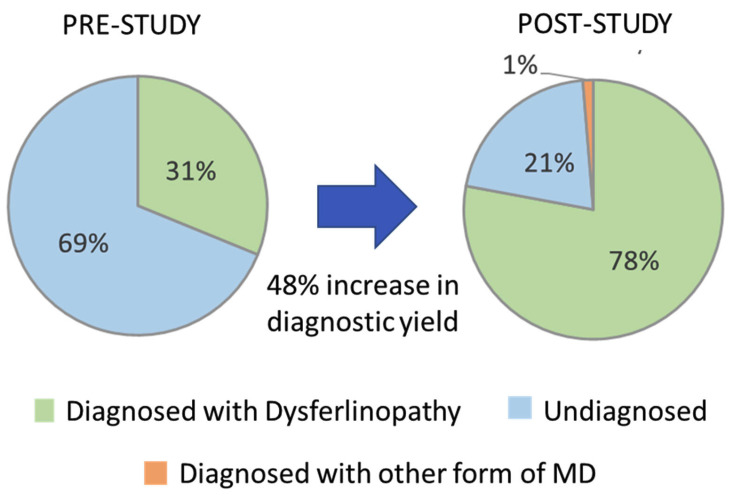
Increase in diagnostic yield.

Prior to the start of this study, 24 (31.2%) of the 77 cases evaluated had two P/LP *DYSF* variants identified based on pathogenicity calls made by other sources and had a predicted diagnosis of dysferlinopathy (cases highlighted in green in the Patient ID column of Supplementary Table S2). The identification of the 21 new *DYSF* variants and the reclassification of *DYSF* variants performed in this study allowed for the identification of 2 P/LP *DYSF* variants in an additional 36 cases (cases highlighted in blue in the Patient ID column in Supplementary Table S2). The likelihood that we were able to identify two P/LP DYSF variants in a case was highly influenced by whether or not a P/LP had already been identified prior to the study. In the 34 cases that had a P/LP *DYSF* variant prior to the study, we were able to identify the second P/LP *DYSF* variant in 88.2% (30) of the cases and achieve a definitive diagnosis. However, for the 19 cases that had one or more *DYSF* variants that were classified as a VUS or that had conflicting calls, only 31.6% (6) were able to obtain a definitive diagnosis of dysferlinopathy. For the four cases in which variants in other genes were identified (JF15, JF126, JF56, and JF371), a confirmed diagnosis was only possible in JF56 due to the identification of a dominant disease-causing variant in *COL6A2*. Therefore, at the end of this study 60/77 cases had a genetically confirmed diagnosis of dysferlinopathy (78%) and 1% (1/77) had a diagnosis of another type of muscular dystrophy (COL6A myopathy), which is an 48% increase in the diagnostic yield for this cohort of individuals (Figure 4).

For the 16 cases that remain unresolved, 6 are unlikely to have dysferlinopathy, with 3 (JF15, JF126, JF371) having zero *DYSF* variants or only one *DYSF* variant classified as a VUS or LB as well as variants in other muscular dystrophy related genes that could explain their phenotype, and 3 cases (JF370, JF392, JF194) having only one *DYSF* variant identified and normal/out of disease range DYSF protein expression. However, it is possible that a *DYSF* variant may have been missed in JF392 and JF194 due to the low splice junction coverage that occurs when whole RNA-Seq is performed instead of targeted RNA-Seq. For the other 10 unresolved cases, 1 case (JF248) has two *DYSF* variants (one classified as LP and the other a VUS) and out of disease range DYSF protein expression; however, one of the *DYSF* variants (c.4794G>T) is a leaky splice site that is associated with out of disease range DYSF protein expression in multiple cases confirmed to have dysferlinopathy and, therefore, this case likely has dysferlinopathy. The other nine cases have disease range/absent DYSF protein levels, which is highly correlated with a dysferlinopathy diagnosis [30]. Those cases with two *DYSF* variants identified (JF198, C155, JF244, JF173, JF255) or one pathogenic *DYSF* variant (JF251) are the most likely to have dysferlinopathy, and continued analysis to reclassify the *DYSF* variants as pathogenic and/or to identify the second *DYSF* variant needs to be performed. For those cases with only one VUS or no identified *DYSF* variant (JF130, JF118, JF340), analysis for other causes of the patient’s muscle weakness should be considered.

## 4. Discussion

Despite the advances in DNA sequencing technology and the greater accessibility of genetic analysis due to the reduction in cost, a substantial number of individuals with probable dysferlinopathy remain without a definitive diagnosis after genetic sequencing. Only 31.2% of the individuals evaluated in this study had a definitive diagnosis of dysferlinopathy prior to subsequent analysis. This highlights the need for additional types of analyses to aid in obtaining a definitive diagnosis. This is especially true for autosomal recessive conditions such as dysferlinopathy, since the identification of two pathogenic variants in *DYSF* can be challenging due to the large number of rare *DYSF* variants identified and the difficulty in determining pathogenicity. As of September 2022, over 5274 *DYSF* variants have been submitted to ClinVar [44]. Many of these variants (40%) are labeled benign or likely benign (2112). For the remaining *DYSF* variants, only 1006 (19%) have been labeled as pathogenic, with 1616 (31%) listed as variants of unknown significance and another 540 (10%) having conflicting calls due to the use of different criteria for determining pathogenicity from the various submitters.

A primary hinderance for making pathogenicity calls is the lack of information for the variant in question. This is especially true for *DYSF,* in which many variants are novel or only seen in a small number of individuals and/or are missense or intronic variants in which the mechanism by which they cause disease is unclear. RNA-Seq is a technique that can provide many types of data such as evidence for pathogenic mechanisms (i.e., aberrant splicing), identification of additional variants (e.g., deep intronic, exonic duplication or deletions, exonic variants missed by traditional sequencing), and information on allelic phasing of variants. This additional information combined with DYSF protein levels and clinical symptoms can often be used to reclassify variants that previously had conflicting calls or were labeled as VUS. The data collected in this study allowed for the ACMG/AMP classification of 86% (95) of the identified variants as either pathogenic, likely pathogenic, or likely benign. While RNA-Seq analysis may not be able to provide additional data to support a pathogenic call in all cases, it can provide relevant data in many instances and should be considered when DNA-based genetic sequencing alone is not sufficient to provide a definitive diagnosis.

While exonic duplication and deletions are not common in *DYSF*, it should be noted that they do occur, as evidenced by the six instances identified in this study. This is consistent with the observation that, of the 1269 *DYSF* variants listed in the LOVD *DYSF* database (as of September 2022), only 25 (2%) intragenic *DYSF* exonic duplications or deletions have been reported (https://databases.lovd.nl/shared/variants/DYSF/unique). However, this is in stark contrast to Duchenne muscular dystrophy, in which 68% of mutations are exonic deletions or duplications [45]. Despite the rarity of such variants in *DYSF*, their evaluation should be included in cases where dysferlinopathy is highly expected and only one P/LP *DYSF* variant has been identified.

Another important observation seen in this study was that most (57%) of the newly identified variants revealed by RNA-Seq were of the type (exonic base pair changes or intronic variants within 20 bp of the beginning or end of an exon) that should have been picked up by the original DNA-sequencing method used. It is unclear why the original DNA sequencing missed these variants. It could be that an older less-robust DNA-sequencing method was originally used. This finding suggests that if dysferlinopathy is highly suspected (especially if the DNA sequencing was performed a number of years ago), a clinician should consider repeating the *DYSF* analysis using a different DNA sequencing method to see if additional *DYSF* variants can be identified to confirm a dysferlinopathy diagnosis.

There are some caveats that should be considered when evaluating the use of RNA-Seq to aid in providing a definitive diagnosis. First, RNA-Seq will not provide additional information in all circumstances. For example, if you have an individual with two missense *DYSF* VUSs, RNA-Seq is unlikely to support allelic phasing, since no nonsense mediated decay would occur. In addition, aberrant splicing will not usually be present unless the missense variant happens to affect splicing, as was seen in the case of five *DYSF* missense variants identified in this study (Table 1, gray highlighting). Predictions of splicing defects for missense and intronic variants can be evaluated using online programs such as SpliceAI (https://spliceailookup.broadinstitute.org/) and TraP (https://trap-score.org/) [46] to determine if RNA-Seq is warranted in order to show the pathological mechanism of a certain variant. Second, the RNA needs to be collected from a source where the gene is highly expressed and that is relevant to the disease state. The *DYSF* gene is an ideal gene for performing RNA-Seq from whole blood because it is overexpressed in blood monocytes [37] and the same spliced transcripts are seen in both the blood monocytes and the main diseased tissue muscle [40]. Data exist from several sources that support that what is happening in blood monocytes is also happening in muscle. The observation that the same *DYSF* spliced variants are seen in RNA from blood monocytes and muscle cells has been experimentally shown by Dominov et al. (2019) [43], who identified the *DYSF* intron 50 variant (c.5668-824C>T) that leads to an aberrant splicing event (Table 1), using RNA isolated from skin fibroblasts cells that were converted into muscle myoblasts using the expression of myoD. This same aberrant splicing event was identified during this study using RNA from blood monocytes of the same individual (JF23 in Dominov et al. is the same individual as C196 in this study). In addition, the dysferlin protein levels detected in blood monocytes and muscle biopsies showed similar levels in the cases in this study that showed aberrant splicing and had dysferlin protein evaluation from both blood and muscle (Supplementary Table S3: JF277, JF308, JF70/C26, JF356, JF250, JF372, JF365). These data provide strong evidence that the splicing pattern observed in blood monocytes and muscle are very similar and supports the use of blood as a non-invasive surrogate for muscle that is relevant to the overall disease state. This is the case for variants in all *DYSF* exons, except for exon 17, which is naturally spliced out in a high percentage of *DYSF* transcripts in the blood [40]. Therefore, if a *DYSF* variant is identified in or effects *DYSF* exon 17, care should be taken when using blood as the source for *DYSF* RNA or protein, as the results will not necessarily represent what is occurring in muscle where exon 17 is present in the majority of mature RNA transcripts.

The 48% increase in diagnostic yield, from 31% to 79%, seen in this study is likely an over estimation of the impact that performing targeted RNA-Seq can have on the overall diagnosis yield in dysferlinopathy, because the cases evaluated for this study were specifically selected on the basis that it was likely that RNA-Seq could provide additional data to aid in pathogenicity classification or identification of additional *DYSF* variants. In addition, the ability to obtain a definitive diagnosis of dysferlinopathy after RNA-Seq is highly dependent on what type of *DYSF* variants you begin with. If you begin with a P/LP *DYSF* variant, our analysis showed that you are more than twice as likely to achieve the definitive diagnosis of dysferlinopathy than if you start out with one or more VUS. An estimate, based on the proportion of cases sampled with different numbers of P/LP/VUS variants identified by DNA sequencing prior to RNA-Seq, is that the diagnostic yield would only double, from 31–59%, if RNA-seq was performed on all cases [47]. Therefore, the increase in diagnostic yield that can be achieved in an unbiased cohort of individuals suspected to have dysferlinopathy using the methods described in this study is much lower than the 79% yield described here.

Clinicians should keep these considerations in mind when determining whether or not to perform RNA-Seq analysis. Nevertheless, this study clearly shows the incredible power that RNA-Seq can have in certain circumstance to provide the information needed to obtain a definitive diagnosis. In addition, this study showed the utility of performing additional DNA-based analyses (e.g., CNV and repeat of DNA sequencing) in order to find additional *DYSF* variants. Therefore, the overall conclusion and recommendations that can be drawn from this study are that clinicians should consider performing additional RNA- or DNA-based evaluations for any cases in which dysferlinopathy is highly suspected based on clinical, genetic, and/or protein evaluations but who do not have two clearly P/LP *DYSF* variants identified after DNA sequencing. These additional analyses could provide the supplemental information needed to lead to a definitive diagnosis, which every patient deserves to have.

## Figures and Tables

**Table 1:** 27 *DYSF* variants that cause splicing errors as determined by RNA-Seq.

*DYSF* Variant (NM_003494)	Consequence of Splicing Error Seen by RNA-Seq (NP_003485.1)	% of RNA Transcripts with Associated Aberrant Splicing
c.855+1delG	p.V286WfsX2	27%
c.857T>A	p.Val286Glu (missense) p.Val286_Arg302del	50–60% 3–5%
c.907-3C>A	p.Met303GlyfsX25 p.Val286GlyfsX25	49% 10%
c.1053+1G>A	p.Arg313ProfsX8 p.His314GlufsX9 p.Val286_Pro351del	5% 10% 7%
c.1171_1180+4dup14 *	p.Met394SerfsX10	100%
c.1180+5G>A	p.Met394SerfsX27 p.Val385TrpfsX5	20% 12%
c.1481-1G>A	p.Glu494GlyfsX16	ND
c.1639-6T>A	p.Gly547AlafsX24	43%
c.2163-2A>G	p.Gln722LeufsX26 p.Gln722ProfsX51	34% 10%
c.2643+1G>A	p.Tyr838_Thr881del	31–60%
c.2643+5G>A	p.Tyr838_Thr881del	32%
c.2810+1G>A	p.Tyr882SerfsX4 p.Trp930_Thr937del	ND
c.2811-2A>C	p.Leu938ArgfsX3 p.Leu938ArgfsX16 p.Leu938AlafsX15	12% 8% 3%
c.2811-20T>G	p.Leu938ArgfsX3	ND
c.3031+2T>C	p.Asn976AlafsX46	37%
c.3031G>C	p.Gly1011Argfs p.Asn976AlafsX46	40% 8%
c.4794G>T	p.K1598N (missense) p.G1547_K1598del	15–18% 26–30%
c.4886+1249G>T	p.Lys1646_Met1647ins59 p.Lys1646AsnfsX13	26% 4%
c.5057+5G>A	p.Gly1660AlafsX36 p.Cys1685SerfsX36 p.Ser1687TyrfsX20	27% 3% 7%
c.5057+5G>T	p.Gly1660AlafsX36 p.Cys1685SerfsX36	45% 8%
c.5429+1G>T	p.Gly178ValfsX17	48%
c.5429G>A *	p.Gly178ValfsX17	100%
c.5503A>G	p.Met1835LeufsX6	46%
c.5526-7T>G *	p.Gly1842_Trp1843insSerSer	95%
c.5668-7G>A	p.Asp1890ValfsX78	29–33%
c.5668-824C>T	p.Asp1890GlyfsX47	17%
c.5768-1G>C	p.Gly1923AlafsX23	25%

* indicates variants that were identified in the homozygous state in this study, which explains why the percentage of splicing is ~100%. The rest of the variants were identified in the heterozygous state. Gray highlighting indicates *DYSF* variants that were previously identified as missense variants, but whose disease mechanism is in part or in whole a splicing error.

**Table 2:** ACMG/AMP classification of *DYSF* variants identified in this study.

*DYSF* Variant (Using NM_003494.3)	Previous Pathogenicity Call (Source)	ACMG/AMP Classification in This Study
c.17T>A	VUS (ClinVar)	VUS
c.89_236del exons 2–3 deletion	Pathogenic (ClinVar)	Pathogenic
**c.125dupT**	**Novel variant**	**Pathogenic**
**c.237_342del** **exon 4 deletion**	**Novel variant**	**Pathogenic**
c.331C>T	Pathogenic [42]	Pathogenic
c.401C>T	VUS/Likely Pathogenic (ClinVar)	Likely Benign
c.509C>A	VUS/Likely Benign/Benign (ClinVar)	Likely Benign
c.533delG	Likely Pathogenic (LOVD)	Pathogenic
c.757C>T	Pathogenic [42]	Pathogenic
c.855+1delG	Pathogenic [42]	Pathogenic
c.857T>A	Pathogenic [42]	Likely Pathogenic
c.863dupA	Pathogenic (LOVD)	Pathogenic
c.865T>C	VUS (ClinVar)	Likely Pathogenic
c.896G>A	VUS (ClinVar, [42])	Likely Pathogenic
**c.907-2774_3873+827del** **duplication of exons 10–35**	**Novel variant**	**Likely Pathogenic**
c.907-3C>A	VUS (ClinVar)	Likely Pathogenic
c.1004G>C	Benign (LOVD)	VUS
c.1053+1G>A	Pathogenic/Likely Pathogenic (ClinVar)	Pathogenic
c.1071delC	Pathogenic (LOVD)	Pathogenic
c.1168G>A	Pathogenic [42]	Likely Pathogenic
c.1171_1180+4dup14	Pathogenic/Likely Pathogenic (ClinVar)	Likely Pathogenic
c.1180+5G>A	Pathogenic [42]	Pathogenic
c.1368C>A	Pathogenic [42]	Pathogenic
c.1392dupA	Pathogenic [42]	Pathogenic
c.1481-1G>A	Pathogenic (ClinVar)	Pathogenic
c.1517C>G	Pathogenic (LOVD)	Pathogenic
c.1639-6T>A	Pathogenic/Likely Pathogenic (ClinVar)	Pathogenic
c.1663C>T	Pathogenic [42]	Pathogenic
c.1668_1669insGTT	VUS (ClinVar)	Likely Pathogenic
c.1834C>T	Pathogenic [42]	Pathogenic
c.1852G>A	VUS/Likely Pathogenic (ClinVar, [42])	Pathogenic
c.1861G>A	Pathogenic [42]	Pathogenic
c.1877T>C	VUS (ClinVar, [42])	Likely Benign
c.1948delC	Pathogenic [42]	Pathogenic
c.1966A>G	VUS/Likely Benign (ClinVar)	VUS
c.2071C>T	Pathogenic (LOVD)	Pathogenic
c.2077delC	Pathogenic (LOVD)	Pathogenic
c.2079C>T	VUS/Likely Benign (ClinVar)	VUS
c.2163-2A>G	Pathogenic [42]	Pathogenic
c.2332C>T	VUS/Likely Benign (ClinVar)	VUS
c.2496_2499delGACA	Pathogenic [42]	Pathogenic
c.2512_3174del exons 25– 29 deletion	Pathogenic (LOVD)	Pathogenic
c.2643+1G>A	Pathogenic [42]	Pathogenic
**c.2643+5G>A**	**Novel variant**	**Likely Pathogenic**
c.2790G>C	VUS (ClinVar)	Likely Pathogenic
c.2810+1G>A	Pathogenic/Likely Pathogenic (ClinVar)	Pathogenic
**c.2811-20T>G**	**Novel variant**	**Likely Pathogenic**
c.2811-2A>C	Pathogenic [42]	Pathogenic
c.2875C>T	Pathogenic [42]	Pathogenic
c.2894G>A	Pathogenic [42]	Pathogenic
c.2929G>A	VUS (ClinVar)	VUS
c.3022G>A	VUS (ClinVar)	VUS
c.3031G>C	Pathogenic (LOVD)	Likely Pathogenic
c.3031+2T>C	Pathogenic [42]	Pathogenic
c.3051dupC	Pathogenic/Likely Pathogenic (ClinVar)	Pathogenic
c.3065G>A	Benign/Likely Benign (ClinVar)	Likely Benign
c.3112C>T	Pathogenic [42]	Pathogenic
c.3113G>A	Pathogenic [42]	Pathogenic
**c.3113G>C**	**Novel variant**	**Likely Pathogenic**
c.3137G>A	Pathogenic [42]	Pathogenic
c.3191_ 3196dupCGGAGG	Benign/Likely Benign (ClinVar)	Likely Benign
c.3444T>A	Pathogenic/Likely Pathogenic (ClinVar)	Pathogenic
c.3445G>A	VUS (ClinVar)	VUS
c.3466T>C	VUS (ClinVar)	VUS
c.3517dupT	Pathogenic/Likely Pathogenic (ClinVar)	Pathogenic
c.3534C>T	VUS, Likely Benign, Benign (ClinVar)	Likely Benign
c.3702T>C	VUS/Likely Benign (ClinVar)	VUS
**c.3703_3843del** **exon 34 deletion**	**Novel variant**	**Likely Pathogenic**
c. 3805dupG	Pathogenic/Likely Pathogenic (ClinVar)	Pathogenic
c.3832C>T	Pathogenic [42]	Pathogenic
**c.3904_4410del** **exons 37–40 deletion**	**Novel variant**	**Likely Pathogenic**
c.3967C>G	VUS/Likely Benign (ClinVar)	VUS
c.3992G>T	Benign/Likely Benign (ClinVar)	Likely Benign
c.4003G>A	Pathogenic/Likely Pathogenic (ClinVar)	Pathogenic
c.4024C>T	VUS/Pathogenic (ClinVar)	Likely Pathogenic
c.4060_4062delTCC	VUS/Pathogenic (LOVD)	Likely Pathogenic
c.4090C>T	Pathogenic (ClinVar)	Pathogenic
c.4360G>T	Pathogenic/Likely Pathogenic (ClinVar)	Pathogenic
c.4408C>T	Pathogenic/Likely Pathogenic (ClinVar)	Pathogenic
c.4439A>C	VUS (ClinVar)	Likely Pathogenic
**c.4509+1586dupG**	**Novel variant**	**VUS**
**c.4526T>G**	**Novel variant**	**VUS**
c.4577A>C	VUS/Likely Benign (ClinVar, [42])	Likely Benign
c.4742G>A	VUS/Likely Benign/Pathogenic (ClinVar)	VUS
c.4756C>T	Pathogenic [42]	Pathogenic
c.4794G>T	VUS (ClinVar)	Likely Pathogenic
c.4886+1249G>T	Pathogenic (ClinVar)	Likely Pathogenic
c.5022delT	Pathogenic/Likely Pathogenic (ClinVar)	Pathogenic
c.5057+5G>A	Pathogenic/Likely Pathogenic (ClinVar)	Likely Pathogenic
c.5057+5G>T	VUS (ClinVar)	Likely Pathogenic
c.5059T>C	VUS (ClinVar)	Likely Pathogenic
c.5159delG	Of the type predicted to cause disease (UMD-DYSF)	Pathogenic
c.5181delA	Pathogenic (LOVD)	Pathogenic
c.5296G>A	VUS/Pathogenic (LOVD)	Likely Pathogenic
**c.5341G>A**	**Novel variant**	**Likely Pathogenic**
c.5429G>A	Pathogenic/Likely Pathogenic (ClinVar)	Pathogenic
c.5429+1G>T	Pathogenic (ClinVar)	Likely Pathogenic
c.5503A>G	VUS (ClinVar)	Pathogenic
c.5509G>A	Pathogenic [42]	Pathogenic
c.5526-7T>G	VUS (ClinVar)	VUS
c.5668-7G>A	Pathogenic [42]	Pathogenic
c.5668-824C>T	Pathogenic [43]	Pathogenic
c.5698_5699delAG	Pathogenic (ClinVar)	Pathogenic
c.5768-1G>C	Likely Pathogenic (ClinVar)	Pathogenic
c.5768_5926del exon 52 deletion	Pathogenic (ClinVar)	Pathogenic
c.5836_5839delCAGC	Pathogenic (ClinVar)	Pathogenic
c.5979dupA	Pathogenic [42]	Pathogenic
c.6038C>G	VUS (ClinVar)	Likely Pathogenic
c.6124C>T	Pathogenic [42]	Pathogenic
c.6196G>A	VUS (ClinVar)	Likely Pathogenic
c.6216delC	Pathogenic (ClinVar)	VUS

(See Supplementary Methods and Supplementary Table S4 for details on how classifications were determined. Variants in bold are novel variants found in this study.

## Data Availability

Not applicable.

## Supplementary Materials

The following supporting information can be downloaded at: https://www.mdpi.com/article/10.3390/jpm13030520/s1, Supplementary Table S1: Neuromuscular Disease (NMD) Gene Panel for Targeted RNA-Seq; Supplementary Table S2: RNA-Seq results; Supplementary Table S3: Clinical data; Supplementary Table S4: The detailed evidence used for the ACMG/AMP classification of the *DYSF* variants identified in the 77 individuals evaluated; Supplementary Material and Methods.
