# Supplementary material for: Utilization of Targeted RNA-Seq for the Resolution of Variant Pathogenicity and Enhancement of Diagnostic Yield in Dysferlinopathy
Source: J Pers Med. 2023 Mar 13;13(3):520. doi: 10.3390/jpm13030520 (PMC10056012; doi:10.3390/jpm13030520)
Supplement: Supplementary file 1 [file jpm-13-00520-s001.zip › Supplementary material and methods.pdf]

Supplementary Materials and Methods for:

Utilization of targeted RNA-Seq for the resolution of variant pathogenicity and enhancement of diagnostic yield in Dysferlinopathy

Authors: Laura Rufibach [1,*,†], Kiera Berger [2,†] , Samya Chakravorty [3,4,†], Sarah Emmons [1], Laurie Long [1], Greg Gibson [2], and Madhuri Hegde [3,5]

TABLE OF CONTENTS

**SUPPLEMENTARY MATERIALS AND METHODS**	**Page Numbers**
1. Patient Enrollment Questionnaire for running ALDA	2-9
2. Targeted RNA-Seq Tiered Analysis Approach	9-12
2.1 RNA-Seq Alignment and Quality Control	9-10
2.1 RNA-Seq Variant Calling	10
2.3 RNA-Seq Splicing Analysis	10-11
2.4 RNA-Seq Allele Expression Imbalance	11
2.5 Allele Expression Imbalance (AEI) Calculation Method	11-12
2.6 RNA-Seq Gene Expression Analysis	12
3. Variant classification methods	12-16
3.1 Pathogenic codes	12-14
3.2 Benign Codes	14
3.3 Rules for combining pathogenic criteria	15
3.4 Rules for combining benign criteria	15-16
4. Supplementary References	16-17

SUPPLEMENTAL MATERIALS AND METHODS

1 Patient Enrollment Questionnaire for the running of ALDA

1. Gender
    - Male
    - Female

2. Parental Consanguinity
    - Yes
    - No
    - Unknown

3. Is there anyone in the patient's with similar symptoms? If so, are their symptoms the same, milder, or more severe than the patient's?
   NOTE: list all family members and how their symptoms compare
    - Brother
    - Sister
    - Father
    - Mother
    - Son
    - Daughter
    - Maternal Uncle
    - Other aunt or Uncle
    - Male first cousin
    - Female first cousin
    - Other

4. Ancestry (select all that apply)
    - Finnish
    - Other Northern European
    - Japanese
    - Not listed
    - Unknown

5. Have any LGMD subtypes or other conditions been ruled out for this patient by genetic testing? NOTE: It is recommended that you initially run the tool without any exclusions
    - Yes

- No
- Unknown

If yes, check all the diseases that apply

☐ 2A ☐ 2B ☐ SG's ☐ 2G ☐ 2H ☐ 2I ☐ 2J ☐ DG's ☐ 2L ☐ 2Q

☐ 1A ☐ 1B ☐ 1C ☐ 1D ☐ 1E ☐ 1F ☐ 1G ☐ 1H ☐ Nonaka/HIBM

☐ Tibal ☐ Becker ☐ DMD Manifesting Carrier ☐ FSH ☐ EDMD

☐ Pompe ☐ Bethlem

6. Age of onset of symptoms

   NOTE: Onset of symptoms should not include high CK without weakness symptoms or subclinical changes e.g. on MRI.

   - <5 years
   - 5-12
   - 13-25
   - >25
   - Unknown

7. Distal Weakness

   - Primarily Distal
   - Comparable to or less than proximal weakness
   - None, or much less than proximal weakness with much later onset
   - Unknown

8. Facial weakness

   NOTE: Examples of this could be difficulty chewing, whistling, making normal facial expression. Also, onset is particularly early in the disease course prior to having severe generalized weakness.

   - Yes
   - No
   - Unknown

9. Cardiac Involvement

   NOTE: Arrhythmia might be related but infarctions probably aren't

   - Yes
   - No
   - Unknown

10. Breathing difficulties
    Does patient have any problems with your breathing that are related to your muscular dystrophy?

    - Yes
    - No
    - Unknown

11. Eyes
    NOTE: Did patient have eye problems (e.g. cataracts, severe myopia, or retinal degeneration) before the age of 20?

    - Affected
    - Unaffected
    - Unknown

12. Central Nervous System (Mental Retardation)
    - Affected
    - Normal
    - Unknown

13. Scapular Winging
    NOTE: If the answer is NO, then you need to determine for how long they have had muscular dystrophy symptoms. If it has been for less than a couple of years, then the answer should be UNKNOWN because we don't know how soon patients start to show scapular winging
    - Pronounced
    - Mild
    - No
    - Unknown

14. Calf Hypertrophy
    Note: Answer PRONOUNCED or MILD if calf muscles were enlarged earlier in the disease course, even if they aren't now

    - Pronounced

- Mild
- No
- Unknown

15. Pain/Cramps

- Cramps/Exercise intolerance (cramping occurs shortly after exercise)
- Pain/Burning (muscles achiness is not associated with exercise)
- No Pain
- Unknown

16. Joint Contractures (early in disease course, if patient currently now has severe generalized weakness)
- Pronounced
- Mild
- No
- Unknown

17. Asymmetry of Weakness/Wasting
- Pronounced (persistent)
- Mild
- No
- Unknown

18. Rate of progression
NOTE: descriptions of each rate can be found below

- Rapid: significant increase in weakness within a few years, causing loss of function and/or walking and/or involvement of other muscle groups beyond the first ones affected.
- Moderate: increase in weakness over several years, but only a slight increase in weakness from year to year. No loss of walking or great difficulty walking within 10 years of onset.
- Slow: very gradual or no decrease in strength for several years following diagnosis. Function appears stable or nearly so.
- Unknown: answer this if a detailed history isn't available, or if patient has only recently experienced symptoms (<3 years) and there has not been noticeable progression.

19. Biopsy: Dystrophic
    NOTE: Does the patient's muscle biopsy show a dystrophic appearance (such as fiber size variation, central nucleation, focal necrosis, fiber replacement by fatty or connective tissue)?
    - Yes
    - No
    - Unknown

20. Biopsy: Inflammation –
    Note: Does patient's biopsy show inflammatory characteristics (e.g., substantial levels of mononuclear cell infiltration?

    - Yes
    - No
    - Unknown

21. Biopsy: Vacuoles/Inclusion Bodies –
    NOTE: Are rimmed vacuoles observed (in substantial numbers) on patient's biopsy?

    - Yes
    - No
    - Unknown

22. CK results
    NOTE: If the patient has more than one CK measurement, use the one taken closest to the onset of the patient's muscle symptoms

    - Unknown/Not Measured
    - <200
    - 200-500
    - 500-2000
    - 2000-7000
    - >7000

23. Does the patient's biopsy show aggregation of desmin or other myofibrillar proteins (conditions with this biopsy appearance are often referred to as a Myofibrillar Myopathy)?
    - Yes
    - No
    - Unknown

24. Prominent Elbow contractures (compared to contractures in other joints)
    - Yes
    - No
    - Unknown

25. Finger contractures
    - Yes
    - No
    - Unknown

26. Toe walking/Achilles tendon tightness
    - Yes
    - No
    - Unknown

27. Foot drop
    Do you trip often or has the doctor ever said you have a foot drop?
    Explanation: This is where you trip over your own foot because you can't pull your foot through the walking motion (lack of dorsiflexion).
    - Yes
    - No
    - Unknown

28. Inability to stand on toes
    - Unable
    - Able
    - Unknown

29. Quadricep Strength (early in disease course if patient now has severe generalized weakness)
    - More affected than other proximal leg muscles
    - Affected comparably to other proximal leg muscles

- Selectively spared compared to other proximal leg muscles
- Unknown

30. Skin Affected?
    NOTE: issues such as blistering or difficulty healing from a cut
    - Yes
    - No
    - Unknown

31. Neck weakness
    - Yes
    - No
    - Unknown

32. Legs or arms more affected
    - Legs
    - Arms
    - Arms and legs comparable
    - Unknown

33. Abnormally flexible joints
    - Yes
    - No
    - Unknown

34. Macroglossia – enlargement of tongue
    - Yes
    - No
    - Unknown

35. Head circumference
    - Abnormally small
    - Abnormally large
    - Normal
    - Unknown

36. Rippling muscles or percussion-induced repetitive
    - Yes
    - No
    - Unknown

37. Nasal or dysarthric speech pattern
- Yes
- No
- Unknown

38. Brain white matter or cerebellar abnormalities seen on MRI
- Yes
- No
- Unknown

39. Cardiac conduction defect
Do you have a pacemaker, or abnormal heart rhythm?
- Yes
- No
- Unknown

40. Dilated Cardiomyopathy
Has your doctor ever told you that you have a "dilated cardiomyopathy"?
- Yes
- No
- Unknown

2. Targeted RNA-Seq Tiered Analysis Approach

2.1 RNA-Seq Alignment and Quality Control

Raw FASTQ files were checked for quality using FastQC (https://www.bioinformatics.babraham.ac.uk/projects/fastqc/) [1]. Reads were not trimmed beyond removal of adapter sequences [2, 3] using Trimmommatic (http://www.usadellab.org/cms/?page=trimmomatic)[1, 4] to prepare for alignment. Human reference genome GRCh38 (NCBI) (https://www.ncbi.nlm.nih.gov/assembly/GCF_000001405.39) and NCBI *Homo sapiens* Annotation Release 106 (https://www.ncbi.nlm.nih.gov/genome/annotation_euk/Homo_sapiens/106/) were obtained from Illumina iGenomes (https://support.illumina.com/sequencing/sequencing_software/igenome.html) and sequenced reads were aligned using the splice-aware alignment program STAR version 2.5.2b (https://code.google.com/archive/p/rna-star/ https://github.com/alexdobin/STAR) in 2-pass mode [5] to improve novel splice junction discovery.

Quality metrics for all samples were obtained by running QoRTs v1.2.42 (http://hartleys.github.io/QoRTs/)[6], and principal component analysis (PCA) on gene expression was performed to check for outlier status based on tissue composition or contamination. We used PCA of the 274 gene expression of the patient samples for quality control which showed better clustering towards whole blood RNA-Seq compared to lower quality and coverage, and 3′-bias for RNA-Seq using CD14+ monocytes or PBMCs (data not shown). Uniquely mapped, non-duplicate read counts for genes and splice junctions were obtained by removing duplicate reads from STAR-aligned BAM files using Picard MarkDuplicates (http://broadinstitute.github.io/picard), converting to FASTQ files, and realigning with STAR using the same parameters as before.

2.2 RNA-Seq Variant Calling

To confirm RNA expression of variants identified by DNA-Seq and check for sequence variants not reported from DNA, we followed the protocol outlined in GATK Best Practices for Variant Calling in RNA (https://software.broadinstitute.org/gatk/documentation/article?id=4067)[7-9]. Filtered VCF files were annotated using ANNOVAR (http://annovar.openbioinformatics.org/en/latest/)[10] and high quality variants in *DYSF* were extracted for evaluation using ACMG-AMP Guidelines for Variant Interpretation[11]. High quality loss of function (LOF) variants and variants with an allele frequency of <5% in the Genome Aggregation Database (gnomAD: https://gnomad.broadinstitute.org/) [12] were also pulled for evaluation from the remaining 273 genes in the panel. All potentially causative variants were manually evaluated using the Integrative Genomics Viewer (IGV: https://software.broadinstitute.org/software/igv/) [13] to ensure they were not a result of mis-mapping or noise. We also used *in silico* prediction algorithms, namely Polyphen2 (Polymorphism Phenotyping v2: http://genetics.bwh.harvard.edu/pph2/)[14, 15], SIFT (Sorting Intolerant From Tolerant: https://sift.bii.a-star.edu.sg/)[16-18], MutationTaster (http://www.mutationtaster.org/)[19], FATHMM (Functional Analysis through Hidden Markov Models: http://fathmm.biocompute.org.uk/)[20], and Transcript-inferred Pathogenicity (TraP: http://trap-score.org/)[18] score for further tentative understanding of the variant pathogenicity.

2.3 RNA-Seq Splicing Analysis

Counts for splice junctions (annotated and unannotated) that overlapped *DYSF* were extracted from STAR output files from all samples. Splice events were considered "annotated" if they matched a known transcript. "Unannotated" events were further analyzed to see if either junction matched an exon/intron boundary in a known transcript. Events fitting this criterion were only kept for analysis if they had read

support totaling more than 5% than that of the matched junction. For events where neither junction matched a known exon/intron boundary, they were still considered for further analysis if the read support was >10% than that of the matched junction. The filtered group of unannotated splice events was further curated by the number of samples each was observed in. If an event had >5% read support in more than half of our control samples, it was automatically eliminated as a potential pathogenic event. This extremely conservative cutoff was chosen because the goal at this stage was merely to narrow the list of splice events undergoing manual evaluation in later steps. Each of the unannotated splice events from the curated list was observed using the Integrative Genomics Viewer, prioritizing the events occurring in just one sample. This led to the identification of 38 pathogenic aberrant splicing events, for which 27 causative variants had been reported from DNA sequencing either as a pathogenic or likely pathogenic variant or VUS or not found in DNA testing but found by RNA-Seq. Expanding the analysis to events in a handful of samples found an additional 3 pathogenic splicing events.

2.4 RNA-Seq Allele Expression Imbalance (AEI)
To evaluate allele expression across *DYSF*, the allele ratio for each individual high confidence single nucleotide variant (SNV) in each sample was calculated by dividing the read count of the lesser-expressed nucleotide (lesser allele) by the total number of reads at the variant position. Read counts for SNVs passing all filters were obtained from the RNA-Seq variant calling VCF file. Each SNV was grouped by the number of PTVs in the sample it belonged to. Because the data did not pass tests for normality or homogeneity of variance, significant differences between groups were calculated using Wilcoxon rank sum tests and p-values were adjusted using the Benjamini-Hochberg method.

2.5 Allele Expression Imbalance (AEI) Calculation Method
Only exonic heterozygous SNVs in DYSF located in constitutively expressed exons, called at >50X per allele and passing all variant quality filters, were considered in the analysis. Samples that were observed to be outliers by gene expression PCA or did not contain any heterozygous SNVs in DYSF were excluded from AEI analysis. Further criteria for sample inclusion was a requirement that the sample contained at least two heterozygous SNVs located more than 150 coding bases apart, to show that the observed AEI is consistent across the entire length of the transcript and that any effect seen is not local to any single variant. A total of 50 samples including 6 controls met the criteria for inclusion. In each sample, allele ratios were calculated for every SNV meeting the stated criteria by taking the lower number of allele-supporting reads divided by the total available reads at that site for Allele A and the greater number of

allele-supporting reads divided by the total site reads for Allele B. In this manner, allele expression is divorced from the concept of “reference” or “alternate” allele. Both AEI and overall gene abundance were correlated with the observation of PTVs in a sample and we attempted to keep the gene abundance observation in the visualization of AEI. Variant call depth is not normalized and varied widely within individuals since depth of coverage is not consistent across all exons, so the calculated allele ratios were instead applied to the overall gene TPM for plotting as a more stable representative of abundance. Each sample is represented by two plotted points showing the average of Allele A and B connected by a line. Error bars represent one standard deviation.

2.6 RNA-Seq Gene Expression Analysis
Non-duplicate read counts for all genes in the panel were obtained from STAR output files. Transcripts Per Million (TPM) Normalization was performed to control for sequencing depth and to make samples directly comparable. Comparisons of gene expression were performed using Welch’s t-test followed by pairwise t-tests with non-pooled SD. P-values were adjusted using the Benjamini-Hochberg method.

3. Variant classification methods

3.1 Pathogenic codes:

PVS1 (varying strengths) - We applied the PVS1 strengths according to the ClinGen Sequence Variant Interpretation (SVI) recommendations for Interpreting the Loss of Function PVS1 ACMG/AMP Variant Criteria [21]. When attributing PVS1 to splice variants or exonic deletions or duplications, proof of a splicing error was based on the RNA-Seq findings shown in Supplementary Table S2. Nonsense variants at or after nucleotide c.6154 and frameshifts that stop on or after nucleotide c.6103 weren’t considered to be undergoing nonsense mediated decay (nmd). The nucleotide numbering is based on *DYSF* transcript NM_003494.4.

PM2 (moderate) – PM2 was applied at the moderate level when the POPMAX filtering allele frequency was ≤0.01%. The POPMAX Filtering Allele Frequencies were obtained from gnomAD v2.1.1 [22]

PM3 (varying strengths) - PM3 strength was assigned according to the SVI Recommendation for in *trans* Criterion PM3 version 1 - (https://www.clinicalgenome.org/site/assets/files/3717/svi_proposal_for_pm3_criterion_-_version_1.pdf). Confirmation that a variant was in *trans* was determined using the

reciprocal AEI ratios found during RNA-Seq (Supplementary Table S2) or through parental testing.

PM4 (moderate) – PM4 was applied at the moderate strength when the protein length changed by ≥6 amino acids

PM5 (varying strength) – PM5 strength was assigned according to the following:

- PM5_moderate - 1 P variant, or 2 LP variants with no benign variation at the residue
- PM5_strong - 2 P or 3 LP variants with no benign variation at the residue

  NOTE: Splicing abnormalities had to have been excluded and variant must meet PP3

PP1 (varying strength) – PP1 (segregation data) was assigned according to the recommendations by the Hearing Loss ClinGen Working group [23] that focused in part on recessive disorders with the slight modification that the strong strength required the segregation to be across at least 2 families:

- PP1_supporting: 1 affected segregation in addition to proband
- PP1_moderate: 2 affected segregations in addition to the proband
- PP1_strong: 3 affected segregations in addition to the proband across ≥ 2 families

PP3 (supporting) - We applied PP3 at the supporting level (*in silico* prediction of pathogenicity) if the CADD and REVEL score were above 25 and 0.7, respectively. REVEL scores [24] were obtained from https://sites.google.com/site/revelgenomics/downloads and CADD scores [25] from https://cadd.gs.washington.edu/snv, using GRCh37-V1.6 model.

PP4 (varying strengths) – PP4 strength was assigned according to the following criteria. The variant must meet PM2 in order for PP4 at any strength to be used. In addition, in all cases the variant in question must be associated with a second P/LP *DYSF* variant.

- PP4_supporting – A supporting strength code was assigned if ALDA showed LGMD2B/R2 as the first or second prediction with at least a medium concordance score and/or clinical data was available either through the study or reported in the literature that indicated progressive weakness over at least 6 months and dystrophic features on biopsy or a myopathic EMG.

- PP4_moderate - A moderate strength code was assigned when the individual met the ALDA and/or clinical criteria described above for PP4 supporting, plus dysferlin protein was absent or in the defined disease range in muscle tissues or monocytes (as detected by western blot or Immunohistochemistry (IHC)).
- No PP4 code was assigned if the studied variant was associated with two pathogenic or likely pathogenic *DYSF* variants that fully explained the dysferlinopathy phenotype

PS3 (varying strengths) – PS3 strong was assigned for splicing and missense variants that showed aberrant splicing that resulted in a frameshift but were not canonical splicing variants (i.e. +/- 1 or 2 intronic positions). PS3 supporting was also used for missense variants in which degradation and mislocalization of the resulting protein has been shown using the *in vitro* assay described by Tominaga et al [26] or when dysferlin protein was absent or in the defined disease range in muscle tissue or monocytes (as detected by western blot or IHC), but clinical data was not available to support assigning a PP4 code.

3.2 Benign codes:

BS1 (strong) – BS1 was applied at the strong level when the POPMAX filtering allele frequency was >0.1%. The POPMAX Filtering Allele Frequencies were obtained from gnomAD v2.1.110 [22].

BP2 (supporting) – We assigned BP2 at the supporting level when a variant was observed in *cis* with a pathogenic variant in *DYSF* or in conjunction with pathogenic variants in another NMD gene which could explain the observed phenotype.

BP4 (supporting) – We applied BP4 score (in silico prediction of pathogenicity) if REVEL score was ≤0.15%. REVEL scores [24] were obtained from https://sites.google.com/site/revelgenomics/downloads

BP7 (supporting) – We assigned BP7 at the supporting level when the RNA-Seq analysis (Supplementary Table S2) ruled out a splicing error for a synonymous variant.

3.3 Rules for combining pathogenic criteria

Pathogenic

- Very strong AND
  - $\geq$ 1 Strong OR
  - $\geq$ 2 Moderate OR
  - 1 Moderate and 1 Supporting OR
  - $\geq$ 2 Supporting
- $\geq$ 2 Strong OR
- 1 Strong AND
  - $\geq$ 3 Moderate OR
  - 2 Moderate AND $\geq$ 2 Supporting OR
  - 1 Moderate AND $\geq$ 4 Supporting

Likely Pathogenic

- 1 Very Strong AND 1 Moderate OR
- 1 Strong AND 1-2 Moderate OR
- 1 Strong AND $\geq$ 2 Supporting OR
- $\geq$ 3 Moderate OR
- 2 Moderate AND $\geq$ 2 Supporting OR
- 1 Moderate AND $\geq$ 4 Supporting OR
- 1 Very Strong AND 1 Supporting

3.4 Rules for combining benign criteria

Benign

- 1 Stand-Alone OR
- $\geq$ 2 Strong

Likely Benign

- 1 Strong and 1 Supporting OR
- ≥ 2 Supporting

SUPPLEMENTARY REFERENCES

1. Wingett, S.W. and S. Andrews, *FastQ Screen: A tool for multi-genome mapping and quality control.* F1000Res, 2018. **7**: p. 1338.
2. Williams, C.R., et al., *Trimming of sequence reads alters RNA-Seq gene expression estimates.* BMC Bioinformatics, 2016. **17**(1): p. 103.
3. Williams, C.R., et al., *Trimming of sequence reads alters RNA-Seq gene expression estimates.* BMC Bioinformatics, 2016. **17**: p. 103.
4. Bolger, A.M., M. Lohse, and B. Usadel, *Trimmomatic: a flexible trimmer for Illumina sequence data.* Bioinformatics, 2014. **30**(15): p. 2114-20.
5. Dobin, A., et al., *STAR: ultrafast universal RNA-seq aligner.* Bioinformatics, 2013. **29**(1): p. 15-21.
6. Hartley, S.W. and J.C. Mullikin, *QoRTs: a comprehensive toolset for quality control and data processing of RNA-Seq experiments.* BMC bioinformatics, 2015. **16**(1): p. 224.
7. McKenna, A., et al., *The Genome Analysis Toolkit: a MapReduce framework for analyzing next-generation DNA sequencing data.* Genome Res, 2010. **20**(9): p. 1297-303.
8. DePristo, M.A., et al., *A framework for variation discovery and genotyping using next-generation DNA sequencing data.* Nat Genet, 2011. **43**(5): p. 491-8.
9. Van der Auwera, G.A., et al., *From FastQ data to high confidence variant calls: the Genome Analysis Toolkit best practices pipeline.* Curr Protoc Bioinformatics, 2013. **43**: p. 11 10 1-33.
10. Wang, K., M. Li, and H. Hakonarson, *ANNOVAR: functional annotation of genetic variants from high-throughput sequencing data.* Nucleic Acids Res, 2010. **38**(16): p. e164.
11. Richards, S., et al., *Standards and guidelines for the interpretation of sequence variants: a joint consensus recommendation of the American College of Medical Genetics and Genomics and the Association for Molecular Pathology.* Genet Med, 2015. **17**(5): p. 405-24.
12. Lek, M., et al., *Analysis of protein-coding genetic variation in 60,706 humans.* Nature, 2016. **536**(7616): p. 285-91.
13. Robinson, J.T., et al., *Integrative genomics viewer.* Nat Biotech, 2011. **29**(1): p. 24-26.

14. Adzhubei, I., D.M. Jordan, and S.R. Sunyaev, *Predicting functional effect of human missense mutations using PolyPhen-2.* Curr Protoc Hum Genet, 2013. **Chapter 7**: p. Unit7 20.
15. Zou, M., et al., *Mutation prediction by PolyPhen or functional assay, a detailed comparison of CYP27B1 missense mutations.* Endocrine, 2011. **40**(1): p. 14-20.
16. Flanagan, S.E., A.M. Patch, and S. Ellard, *Using SIFT and PolyPhen to predict loss-of-function and gain-of-function mutations.* Genet Test Mol Biomarkers, 2010. **14**(4): p. 533-7.
17. Ng, P.C. and S. Henikoff, *SIFT: Predicting amino acid changes that affect protein function.* Nucleic Acids Res, 2003. **31**(13): p. 3812-4.
18. Gelfman, S., et al., *Annotating pathogenic non-coding variants in genic regions.* Nat Commun, 2017. **8**(1): p. 236.
19. Schwarz, J.M., et al., *MutationTaster evaluates disease-causing potential of sequence alterations.* Nat Methods, 2010. **7**(8): p. 575-6.
20. Rogers, M.F., et al., *FATHMM-XF: accurate prediction of pathogenic point mutations via extended features.* Bioinformatics, 2018. **34**(3): p. 511-513.
21. Abou Tayoun, A.N., et al., *Recommendations for interpreting the loss of function PVS1 ACMG/AMP variant criterion.* Hum Mutat, 2018. **39**(11): p. 1517-1524.
22. Whiffin, N., et al., *Using high-resolution variant frequencies to empower clinical genome interpretation.* Genet Med, 2017. **19**(10): p. 1151-1158.
23. Oza, A.M., et al., *Expert specification of the ACMG/AMP variant interpretation guidelines for genetic hearing loss.* Hum Mutat, 2018. **39**(11): p. 1593-1613.
24. Ioannidis, N.M., et al., *REVEL: An Ensemble Method for Predicting the Pathogenicity of Rare Missense Variants.* Am J Hum Genet, 2016. **99**(4): p. 877-885.
25. Rentzsch, P., et al., *CADD: predicting the deleteriousness of variants throughout the human genome.* Nucleic Acids Res, 2019. **47**(D1): p. D886-D894.
26. Tominaga, K., et al., *4-Phenylbutyrate restores localization and membrane repair to human dysferlin mutations.* iScience, 2022. **25**(1): p. 103667.
